# Influence of Lime and Volcanic Ash on the Properties of Dune Sand as Sustainable Construction Materials

**DOI:** 10.3390/ma14030645

**Published:** 2021-01-30

**Authors:** Faisal I. Shalabi, Javed Mazher, Kaffayatullah Khan, Muhammad Nasir Amin, Alaa Albaqshi, Abdullah Alamer, Ali Barsheed, Othman Alshuaibi

**Affiliations:** 1 Department of Civil and Environmental Engineering, College of Engineering, King Faisal University (KFU), Al-Ahsa 31982, Saudi Arabia; kkhan@kfu.edu.sa (K.K.); mgadir@kfu.edu.sa (M.N.A.); 214020718@student.kfu.edu.sa (A.A.); 214010919@student.kfu.edu.sa (A.A.); 214110860@student.kfu.edu.sa (A.B.); 215018421@student.kfu.edu.sa (O.A.); 2 Physics Department, College of Science, King Faisal University (KFU), Al-Ahsa 31982, Saudi Arabia; jkhan@kfu.edu.sa

**Keywords:** dune sand stabilization, cement and volcanic ash, CBR, compressive strength, young’s modulus, sustainability, construction materials

## Abstract

This study focused on evaluating dune sand stabilized with lime and volcanic ash as base course materials in engineering construction. Dune sands are found in Saudi Arabia in huge quantities. Due to the high demand for construction materials, this makes them highly suitable for construction. A testing program was designed to investigate the effect of adding different percentages by weight of lime (L: 0, 2, 4, and 6%) and volcanic ash (VA: 0, 1, 3, and 5%) on the engineering properties of the stabilized mixture. Unconfined compressive strength (UCS) and California bearing ratio (CBR) tests were conducted. In addition, Raman spectroscopy and laser-scanning microscopy (LSM) tests were performed to explore the chemical characteristic, packing, and structure of the mixture. The results showed that the UCS, CBR, and the Young’s modulus (E_s_) of the treated dune sand increased with the increase in percentage of both stabilizers. Furthermore, LSM images of mortar blended with intermediate L-to-VA blend ratio ≈0.55 (L: 6% and VA: 5%) exhibit compact packing of sand grains, indicating strong adhesion and higher cementing value. The results of the study are promising and encourage using the treated dune sand in engineering construction even with a low percentage use of lime (2%) and volcanic ash (1–3%) as stabilizers.

## 1. Introduction

Vast growth in industry and population necessitate searching for more and different resources of good quality earth materials for construction work. The extensive use of quarried construction materials for the construction of building and roads have led to severe damage to the environment and increased pollution levels. Governments and environmental agencies place colossal pressure on the construction industry to look for new materials and assimilate sustainability. Extensive research works were carried out to serve sustainability and to stimulate construction process and development. Most of the research work directed toward using different additives to improve the engineering properties of materials not meeting the required standards. In arid regions where dune sands are abundant in huge quantities, many researchers have investigated their use as construction materials. Since dune sands are almost classified as SP or SP-SM according to USCS (A-1 to A-3 according to AASHTO classification) [1], and according to the World Road Association, they are suitable in construction works if they are properly stabilized and treated [2]. Over the last few decades, dune sands were investigated and utilized in construction purposes after stabilization with different additives to improve their geotechnical properties and to meet engineering requirements and specifications. Many researchers concluded that the dune sands located in various world sites almost possess similar physical properties and characteristics [3,4].

Many other studies have been conducted on dune sands to explore their characteristics. Studies on chemical and mineralogical compositions showed that dune sand is mainly composed of quartz [3,4,5]. Studies on the physical properties indicated that dune sands fall in a narrow range of grain sizes (0.08–0.40 mm) [6,7]. For specific gravity of dune sands from different places in the world, the range was found to be wide, ranging between 2.44 (sands from Egypt) [8] to 2.87 (sands from India) [3]. Morphological studies showed that dune sands particles are almost rounded in shape with flat cleavage planes and conchoidal breakage arrangements [3,5,9]. Early attempts to assess dune sand performance in construction was performed by Khan [10] by studying samples from the Libya desert and by Sanad and Bindra [11] by analyzing samples collected from Saudi Arabia. More extensive and focused studies were started to explore the engineering properties of dune sands and derive conclusions based on experimental work.

Many researchers performed several studies for improving the mechanical and microstructural properties of dune sand used in road construction by incorporating a different binder such as cement, lime, ground granulated blast furnace slag, municipality waste, fly ash, silica fume, metakaolin, bentonite, etc. [12,13,14,15,16,17,18,19,20]. Wahab and Asi [12] used lime and Portland cement to accelerate the curing process of dune sand collected from the Eastern part of Saudi Arabia, treated with emulsified asphalt. The results showed an improvement in shear strength and resistance of the treated sand. Mohamedzein et al. [13] evaluated the use of municipal solid waste incinerator ash to stabilize Oman dune sand for geotechnical engineering works. The results of the tests showed a considerable increase in shear strength parameters and UCS of the treated sand. Wayal et al. [14] investigated the effect of using bentonite and lime to stabilize dune sand collected from Rajasthan, India for uses in geotechnical engineering work. The results showed a substantial increase in the UCS of the stabilized sand by adding 3% lime and 15% bentonite. Rabbani et al. [15] investigated the influence of ground granulated blast furnace slag (GGBFS) and lime on the geotechnical properties of dune sands of Iran for possible usage in roadways and railways projects. The results showed significant improvement in the CBR and UCS of the treated sand. Experimental tests on Indian dune sands stabilized with waste crumb rubber showed an increase in friction angle from 30 to 35 by using 25% of waste rubber [16]. Lahlih and Ahmed [21] used sulfonated urea, formaldehyde, and melamine to stabilize dune sand. The results showed a significant increase in the compressive strength of the dune sands even at very low percentages of stabilizers. Homauoni and Yasrobi [22] used poly methyl and polyvinyl to stabilized dune sand for road construction in Iran. The results showed an increase in the shear strength of the stabilized sand in the dry state and a reduction in CBR value for the saturated state compared to the dry state. The results also showed that the optimum polymer content for best effect was 3%. Dune sand from Djelfa-Algeria was stabilized by different percentages of Portland cement to improve its mechanical performance for usage in roads construction. The results of the tests showed improvement in compressive strength, tensile strength, and compressibility of the stabilized sand [17]. Querol et al. [23] and based on an experimental study performed on cement-stabilized dune sand collected from the western part of Saudi Arabia, found a strong relationship between the CBR value (as a measure of bearing capacity) and cement content.

Due to the current trends in infrastructure development, the demand for construction material is also increasing. Nowadays, the research is focused on using locally available materials for the construction of the road network due to their economic and sustainability benefits. Dune sands are available in huge quantities all over Saudi Arabia, and therefore, their uses in the construction industry can be thrifty. The locally available due sand is an attractive material to be used for the construction of roads and foundations. However, due to the low bearing capacity of these soil, it is necessary to improve their properties by using different stabilizers [24,25,26]. In addition, the locally available pozzolan called volcanic ash is also available in large quantities in the western part of the country as natural materials. Comparative to commercially available materials, volcanic ash could be a viable and economical material to be used as a stabilizer.

Therefore, in this study, lime and locally available volcanic ash (VA) were used as stabilizing materials to improve the engineering properties of the dune sand for its potential use as a base course material for roads and foundations construction. Different percentages of lime (0%, 2%, 4%, and 6%, of the dry weight of the sand) and of volcanic ash (0%, 1%, 3%, and 5%) and their blends were selected. Initially, all materials were analyzed and characterized by performing the different conventional tests. In the second step, mechanical properties such as UCS, Young’s modulus, and CBR tests were performed for all samples with and without lime and volcanic ash and its blends. In the third step, the effect of optimized quantities of lime and VA on the improvement of microstructure was investigated by performing laser scanning microscopy and Raman microscopy analysis. Finally, useful and practical relationships were developed between the UCS, E_s_, and CBR value as a measure of the bearing capacity of the stabilized dune sand for practical use in roadways and foundations construction.

## 2. Materials Used

### 2.1. Dune Sand

**Figure 1:**
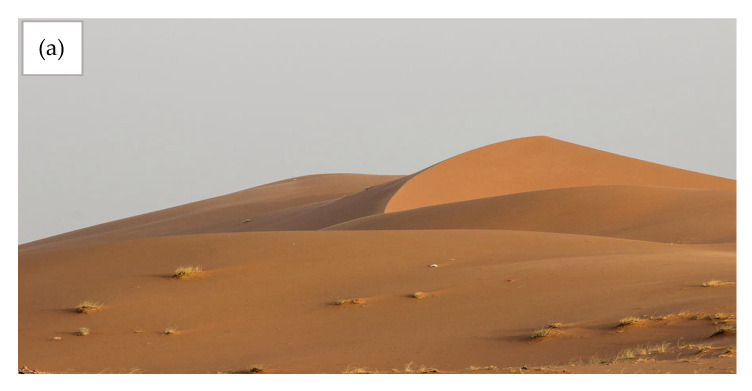

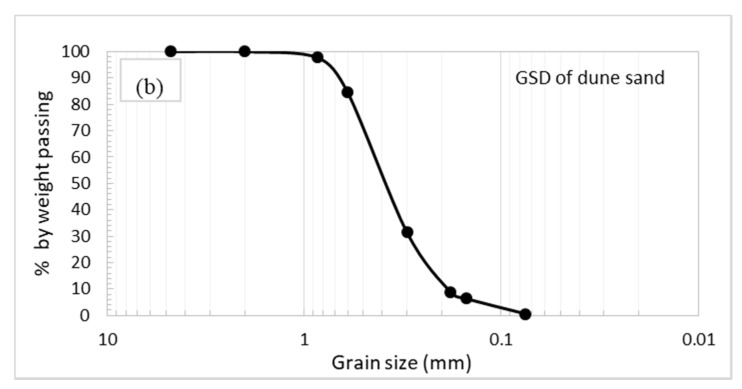
(**a**) Dune sand in the eastern part of Saudi Arabia, and (**b**) grain size distribution of the dune sand.

Dune sand used in this study was collected from one of the stockpiles of construction materials located along Dammam road (the eastern region of Saudi Arabia). Figure 1a shows an aspect of the dune sand in the eastern part of Saudi Arabia. According to the Unified Soil Classification System- ASTM D 6913 and ASTM D2487-17 [27,28], the sand is classified as SP (poorly graded sand), while according to the AASHTO system, AASHTO M145-82 [29], it is classified as A3 (non-plastic fine sand). Figure 1b shows the grain size distribution of the dune sand, which mostly falls in the range of 0.1–0.8 mm (with a medium grain size of D_50_ = 0.4 mm). Table 1 summarizes the physical properties and classification of the dune sand according to standards.

### 2.2. Volcanic Ash (VA)

Basaltic volcanic ash was collected from the western part of Saudi Arabia, near the city of Jeddah. Millions of years ago, the western part of the country was subjected to volcanic activities, which led to the formation of basaltic flows called “Harrat” which cover an area of about 90,000 km^2^ [31]. Table 2 shows the physical and chemical properties of the used volcanic ash. In this table, it can be seen that Silicon Oxides (SiO_2_), Aluminum Oxides (Al_2_O_3_), and Ferric Oxide (Fe_2_O_3_) are the most abundant oxides that form the chemical composition of the used volcanic ash with 73.6% as a total sum by weight.

**Figure 2:**
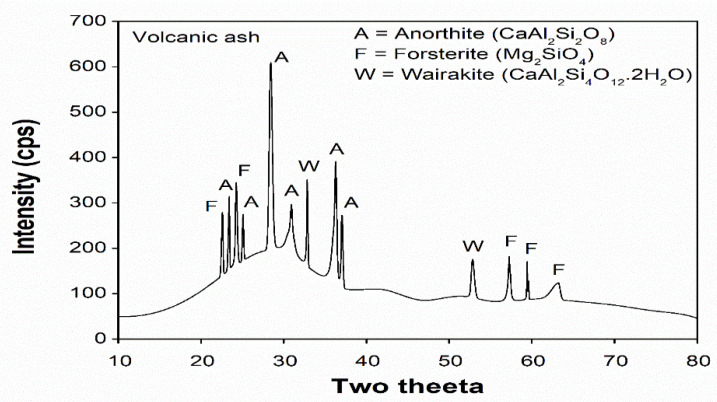
X-ray diffraction analysis of powder sample of volcanic ash (VA).

X-ray diffraction analysis of the volcanic ash powder sample was performed using Rigaku MiniFlex II and XRD intensity peaks as shown in Figure 2. The XRD peaks show the presence of both crystalline and amorphous phases. The main crystalline phases present in the VA are anorthite and forsterite. While on the other hand, the wairakite phase is calcium-based zeolite and the major amorphous phase present in the volcanic ash. According to GSD analysis, D_90_ and D_50_ of the used ash are 25 μm and 3 μm, respectively [31].

### 2.3. Hydrated Lime

The hydrated lime used in this study was procured from a Saudi lime factory. The hydrated lime has a purity level of Ca(OH)_2_, a minimum of 90%, and the particle size analysis shows that 90% of the grain size is less than 90 micron. When lime is added to silicon and aluminum oxides (within soils or added volcanic ash) with the presence of water, pozzolanic reactions occur and lead to forming calcium silicate hydrate (C-S-H) and calcium aluminate hydrate (C-A-H) gels that, after crystallization, will substantially contribute to connecting the soil particles to a relatively strong structure [15].

### 2.4. Water

Water used in the testing program was tapped water. According to AASHTO T 26 specifications [32], it has less than 1000 ppm of chloride (CL^−2^) and less than 3000 ppm of sulfates (SO_4_^+2^).

## 3. Experimental Program and Methodology

### 3.1. Physical and Engineering Properties Testing Program

**Figure 3:**
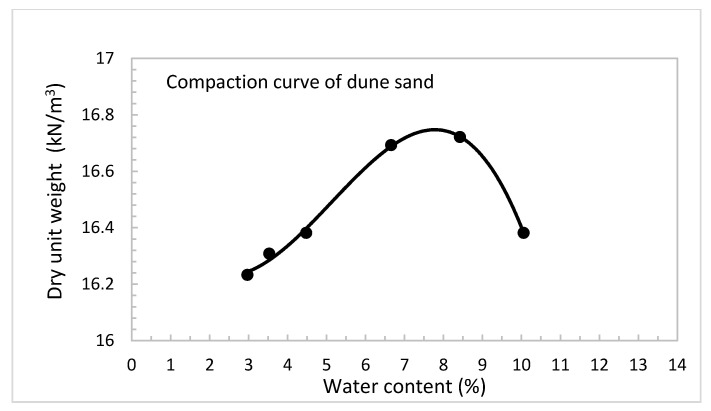
Standard proctor compaction curve of dune sand.

An experimental program was performed to attain the outcomes of this study. The program concentrated on the investigation of the engineering properties and behavior of the stabilized dune sand using various percentages by weight of hydrated lime (0, 2, 4, and 6%) and volcanic ash (0, 1, 3, and 5%) mixed at the maximum unit weight and optimum moisture content of dune sand samples compacted by the standard Proctor test, ASTM D698-07 method A [33]. The preliminary tests considered in this study are the UCS based on ASTM D2166-85 [34] and the CBR based on ASTM D1883-07 [35]. The samples of both CBR and UCS were tested after a curing time of 28 days. After casting, the treated dune sand samples were firmly sealed by thin plastic film and maintained at room temperature of around 22 °C to keep the moisture content constant. Table 3 shows the performed tests at various percentages of lime and volcanic ash. Figure 3 presents the standard Proctor compaction curve of the dune sand with a maximum unit weight of 16.8 kN/m^3^ and optimum water content of 7.8%.

### 3.2. Spectroscopy and Microscopy Testing Program

A testing program was designed to investigate the morphologic, cement chemistry, and pozzolanic reactions between sand, volcanic ash, and lime. The hydration of lime and volcanic ash is performed in different ratios, as shown in Table 4. All measurements were performed after a fixed aging duration of 90 days.

#### 3.2.1. Raman Spectroscopy

Macro Raman spectra were recorded from Horiba Labram (Evolution) spectrometer using a He-Ne laser excitation source (633 nm, wavelength). The spectrometer was calibrated by a Si standard using the Raman peak at 520.7 cm^−1^. All Raman spectra were recorded using the confocal microscopic method keeping a hole size fixed at 50 A.U. (1 airy unit = 1.22 × λ/numerical-aperture) and a spectral accuracy of 0.5 cm^−1^.

#### 3.2.2. Laser Scanning Microscopy

We have deployed the laser scanning microscopy (LSM) method with confocal data acquisition capabilities to perform the morphological studies of sand, lime, ash, and their mixtures in the various ratios under consideration. We collected the morphologies using both the 50× and 5× objectives lenses for high resolution imaging and large area imaging respectively. The scan-areas of 200 μm and 2 mm were used for the two said morphologies. All the imaging data were collected by deploying a confocal hole of a size of ~1 A.U. and 402 nm diode laser for the sample illumination.

## 4. Results and Discussion

### 4.1. Raman Spectroscopy

Raman spectroscopy is a simple and effective technique to explore cement chemistry [36,37]. The active phase blends of cement show higher pozzolanic activity and has been extensively explored in the recent scientific literature of cement mortars [38,39,40,41,42]. The hydrations of lime and ash blends have shown higher cementing strengths due to the formation of many pozzolanic reaction compounds [43,44]. Volcanic ash is known to comprise of active phase constituents of siliceous and aluminous pozzolans such as nanoporous zeolites (aluminum silicates), nanoporous silica, iron-oxides, etc. The pozzolans on hydration with lime in the cementing mortars undergo the formation of calcium silicate hydrates represented as C-S-H in geochemical notation [45]. It should be noted that the pozzolans have no cementing value unless mixed with lime in the presence of water. However lime hydration with pozzolan produces C-S-H compounds, represented by a long duration of pozzolanic reaction Equation (1) below, which provides the required binding strength to the cementing mortars [44].

(1) C−H+S−H→C−S−H

where, the industrial notations, *C*−*H* and *S*−*H* represent calcium hydroxide $Ca{\left(OH\right)}_{2}$ and silicate hydrate $H_{4}SiO_{4}$, which is a silica-based pozzolan. The reaction product, *C*−*S*−*H*, is a pozzolanic reaction compound $CaO\cdot SiO_{2}\cdot 3H_{2}O$. The effect of the *C*−*S*−*H* formation on the mortar strengths has been explained in detail for lime hydration in the recent literature [43,46].

**Figure 4:**
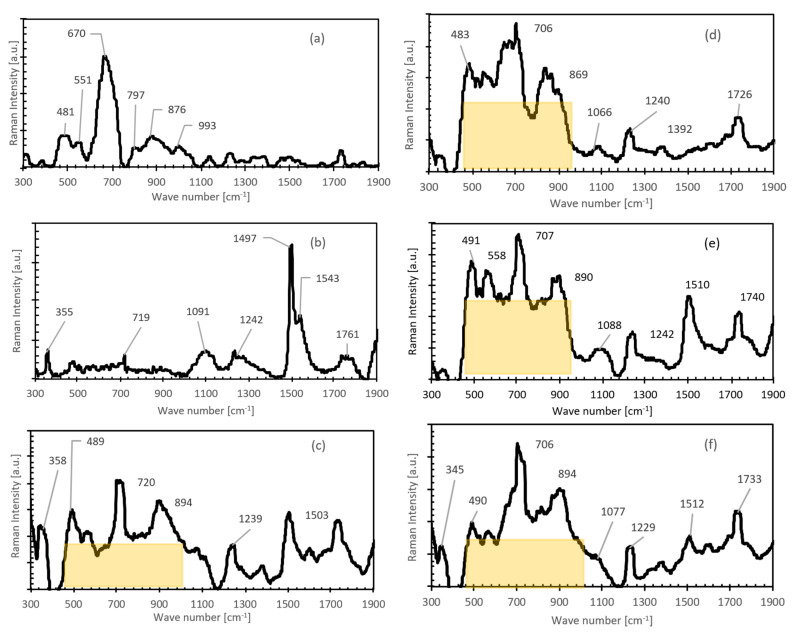
Raman spectra of volcanic ash and pure lime are shown in the bottommost spectra (**a**) and (**b**), respectively. Remaining Raman spectra represent aged cementing mortars prepared in different ratios of lime/ash; 0.86 in Figure (**c**), 0.67 in Figure (**d**), 0.55 in Figure (**e**), and 0.40 in Figure (**f**), arranged in the decreasing quantity of lime content in the mortar. Spectral positions of the major Raman peaks are marked and labeled adjacent to the peak in the units of cm^−1^.

The Raman spectra in Figure 4a,b show the properties of volcanic ash and lime, respectively. The volcanic ash spectrum in Figure 4a has six broad peaks positioned at 481, 551, 670, 797, 876, and 993 cm^−1^ representative of different pozzolans present in volcanic ash. The peak at 481 cm^−1^ is broad and represent bending of various Si-O-Si linkages in the silicates and silica [45]. The second peak at 551 cm^−1^ originates from the Al-O-Al stretching mode of a C_3_A-type pozzolan [47]. Slowly hydrating pozzolan (C_4_AF) peak is also detected at 670 cm^−1^ in the Raman spectrum of volcanic ash indicating the presence of calcium iron aluminates (Ettringite phases) in the pozzolan [48,49]. In addition, the 670 cm^−1^ peak also represents iron (III) oxide minerals (Magnetite) presence [50]. However, the presence of aluminates are further confirmed by the 797 cm^−1^ peak of the AlO_4_ vibration [47]. Another slowly hydrating pozzolan peak is detected at 872 cm^−1^ corresponding to the C_2_S-type silicate (Belite phase) present in the ash [48]. The presence of slowly hydrating pozzolans indicate that the volcanic ash used in this study requires a long cementing time period. The last peak at 993 cm^−1^ also indicates the presence of aluminum silicates (Andalusite) in volcanic ash [50,51].

As shown in Figure 4b, the Raman spectrum of the lime sample confirms the typical characteristic vibrations of calcium hydroxide phase in between 1497–1761 cm^−1^ and portlandite modes at 355 and 719 cm^−1^ [52,53]. The hydration of lime and volcanic ash results are shown in Figure 4c–f with a decreasing content of lime in the blend. The lime to blend ratio is reduced from 0.86 to 0.40 systematically. Owing to the onset of pozzolanic activity in the hydrating blend, we can observe a series of pozzolanic reaction compounds of hydrates of calcium-silicates and -aluminates, indicated by a broad band (yellow box) of Raman peaks in between 450–950 cm^−1^. The pozzolanic activity of the blend and thus the binding performance of the blend are directly related to the size of the yellow box, which signifies the presence of new pozzolanic reaction compounds. The box area is found to increase with the increasing content of ash, pointing towards the requirement of silicates and aluminates to complete the pozzolanic reactions. One can see that the largest number of pozzolanic reaction compounds form at the lime-to-blend ratio ≈0.55. Nevertheless, on further decreasing the ratio ≈0.44, the pozzolanic activity decreases, indicating a deficiency of lime, which is required to complete the reaction.

### 4.2. Laser Scanning Microscopy

**Figure 5:**
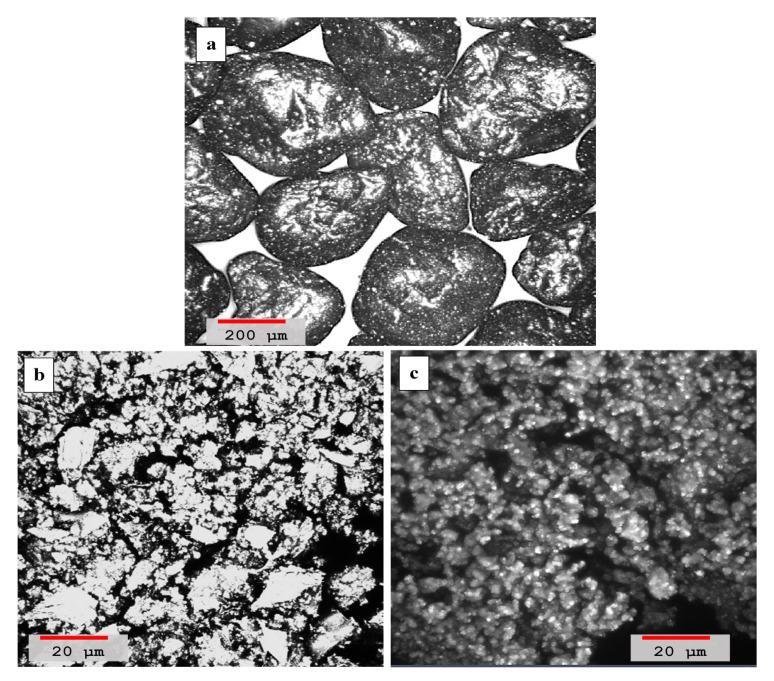
(**a**) Laser scanning microscopy image of grains of sand dunes. The red colored scale-bar in the left shows ≈200 μm length. (**b**) Laser scanning microscopy image of flacks of volcanic ash. The red colored scale-bar in the left shows ≈20 μm length. (**c**) Laser scanning microscopy image of the lime powder. The red colored scale-bar in the right shows ≈20 μm length.

Microscopic images of grains of sand dune particles, volcanic ash, and lime are shown in Figure 5a–c, respectively. Microscopic images of the hydrated samples of the cementing mortars are recorded after 90 days of aging at different lime-to-ash ratios. The long aging duration of blends has been deliberately chosen to fully complete the pozzolanic reaction because the slowly hydrating pozzolan phases (C_2_S and C_4_AF) present in the blends are also proven from the Raman studies.

**Figure 6:**
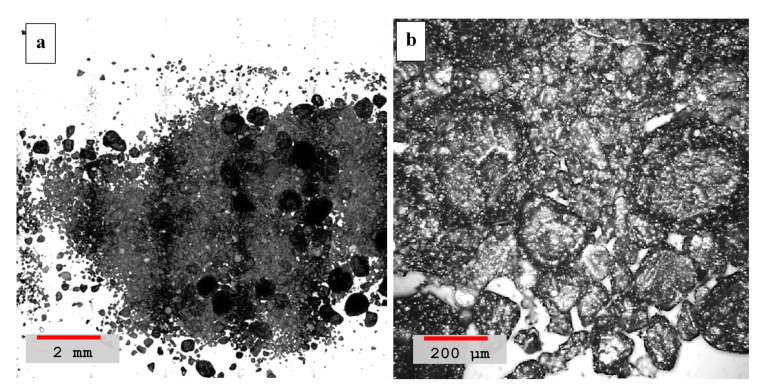
(**a**) Laser scanning microscopy images of hydrated volcanic ash blended mortar with sand, sample *#a* in Table 4, recorded at lower 5× magnification image (red color scale-bar shows ≈2 mm length). (**b**) Morphological image of the sample at a higher magnification of 50× with scale-bar showing ≈200 μm length.

The laser scanning microscope analysis performed on hydrated volcanic ash mortar with sand is shown in Figure 6a,b, both at lower and higher magnifications, respectively. The higher magnification image, Figure 6b, clearly shows a negligible cementing effect since very little or no interaction is observed in between the grains of the sand dune. The individual grains remain separated from each other with almost no interaction manifesting the absence of pozzolanic reaction compound formation. Moreover, the morphology of the hydrated blend is also phase segregated and the ash particles look like flaky structures with sharp edges while the sand dune particles are more distinct with rounded granular structures. The images confirm that both the phases have no binding interaction and thus the presence of pure ash in sand dune mortar has no cementing value.

**Figure 7:**
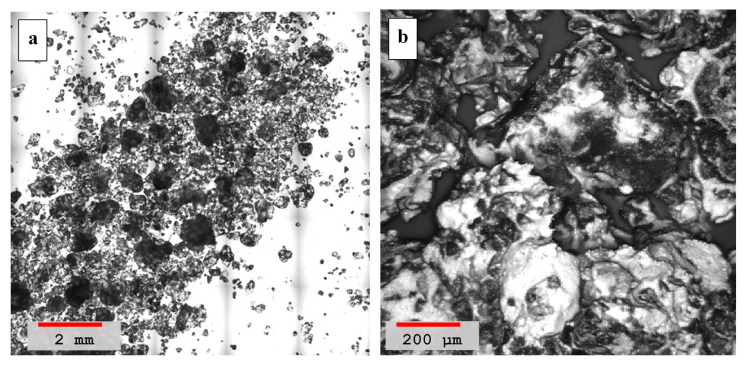
(**a**) Laser scanning microscopy image of hydrated mortar containing pure lime and sand, sample *#b* in Table 4, recorded at a lower 5× magnification image (red color scale-bar shows ≈2 mm length). (**b**) Morphological image of the sample at a higher magnification of 50× with a scale-bar showing ≈200 μm length.

Hydrated lime mortar’s surface morphology is depicted in Figure 7, the wide area scan is shown in Figure 7a, and the high-resolution scan is shown in Figure 7b. Both images show that the adhesion of the mortar grains are not very strong owing to a large number of voids present in between grains. The wider scan, Figure 7a, describes that only some part of the mortar forms bigger lobes due to the lime related weak binding. The high resolution image, Figure 7b, also show some granular rounded edges indicating that some sand dune grains are separated from other grains, forming weaker clusters. The presence of a large number of voids and weak bonding between sand grains observed in lime-sand mixtures is mainly attributed to the formation of a weak compound due to the presence of lime only as compared to mixtures having both lime and volcanic ash.

**Figure 8:**
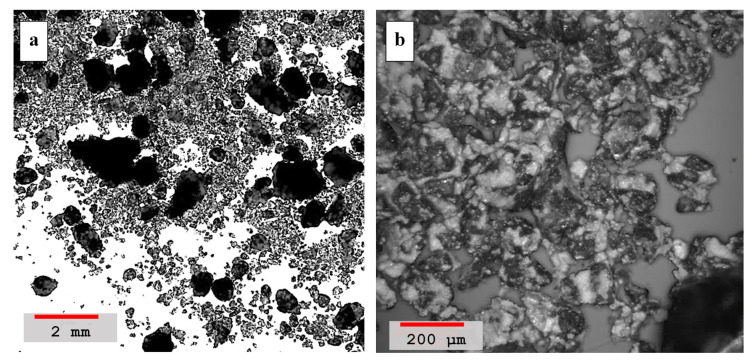
(**a**) Laser scanning microscopy images of hydrated and blended mortar containing the lime-to-ash ratio ≈0.67, sample *#d* in the Table 4, recorded at a lower 5× magnification image (red color scale-bar shows ≈2 mm length). (**b**) Morphological image of the sample at a higher magnification of 50× with scale-bar showing ≈200 μm length.

LSM images of the blended mortar with a lime-to-blend ratio of 0.67 are shown in Figure 8a,b. It is clear from the wide area scan, as shown in Figure 8a, that an increasing amount of lumping behavior is observed among the sand dune grains of the mortar due to the presence of the pozzolanic reaction compounds, which are acting as the binding agents. The darker regions in the image show bigger and stronger clusters that are difficult to separate. A darker contrast of the cementing mortar in the microscopic images is indicative of the formation of C-S-H compounds [48]. Similarly, the high resolution image, Figure 8b, depicts larger-sized clusters with good adhesion between grains and the lime-ash blend indicating the formation of pozzolanic compounds. Moreover, the binding of the grains is stronger than before since no distinct granular edges are observed. In some regions the distinct granular edges apparently indicate not very strong binding due to incomplete pozzolanic reactions. Interestingly, the incomplete pozzolanic reaction is also confirmed in this composition by the preceding Raman studies due to a deficiency of pozzolans.

**Figure 9:**
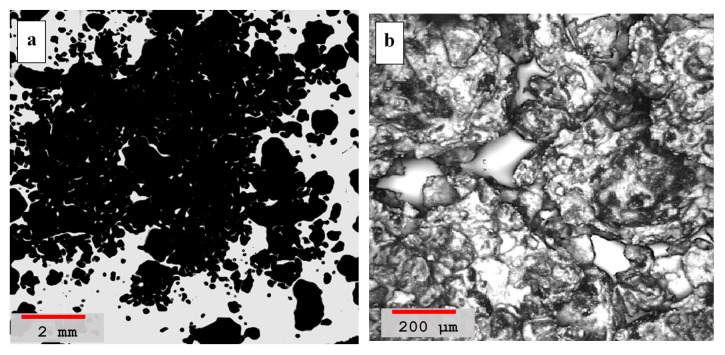
(**a**) Laser scanning microscopy images of hydrated and blended mortar containing a lime-to-ash ratio = 0.55, sample *#e* in Table 4, recorded at a lower 5× magnification image (red color scale-bar shows ≈2 mm length). (**b**) Morphological image of the sample at higher magnification of a 50× with scale-bar showing ≈200 μm length.

In the hydrated and lime-to-blended mortar ratio ≈0.55, all grains are very well connected forming larger lumps due to increased binding properties, as shown in Figure 9. The wide area scan, Figure 9a, shows large clusters bonded strongly with each other and appear in the dark contrast color indicating that the grains are bonded by the pozzolanic reaction compounds. Similarly, the high resolution image, Figure 9b, also depicts void-free morphology of strong cementing mortar. Moreover, the sand-dune’s grains are also not distinct in the image indicating that all the granular boundaries are very well soaked in the binder reaction compounds, forming singular and large solidifications. Thus, the microscopic results are in a perfect overlap with the Raman results, signifying the high density of pozzolanic reaction compounds at a lime-to-blend ratio of 0.55.

### 4.3. Unconfined Compressive Strength (UCS)

**Figure 10:**
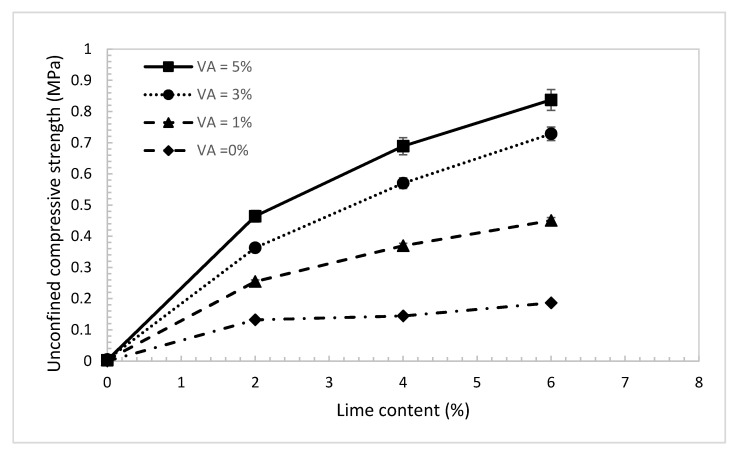
Unconfined compressive strength of the treated dune sand vs. different lime content.

**Figure 11:**
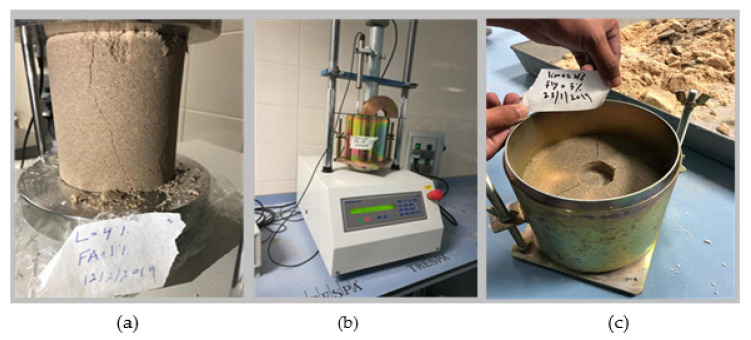
(**a**) UCS-test, (**b**) CBR test, and (**c**) CBR-tested sample.

**Figure 12:**
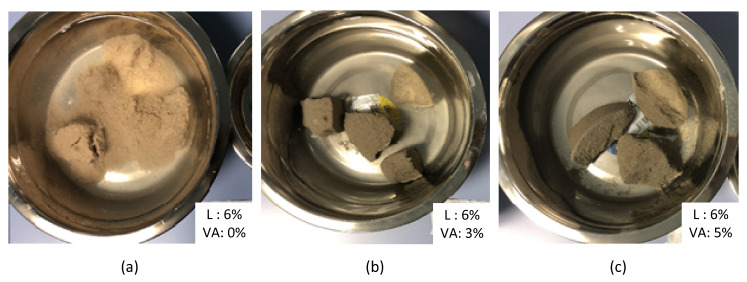
Samples of stabilized dune sand soaked in water. (**a**) L: 6%, VA: 0%, (**b**) L: 6%, VA: 3%, and (**c**) L: 6%, VA: 5%.

The results of UCS of the treated dune sand with lime content and for different percentages of volcanic ash are shown in Figure 10. The tests were conducted at the optimum water content and maximum dry density of the dune sand prepared according to the standard Proctor compaction test (Method A). The size of the tested samples was 102 mm in diameter and 116 mm in height, as shown in Figure 11a. The sand was mixed with different percentages by weight of lime (0, 2, 4, and 6%) and volcanic ash (0, 1, 3, and 5%). The samples were sealed with thin plastic sheets and cured for 28 days at a room temperature of 22 °C. The results in Figure 10 show that with the absence of volcanic ash, UCS increases from almost zero at a lime content of 0% to 0.12 MPa at a lime content of 2%, and for a lime content above 2%, there is almost no increase in the strength of the treated sand. As the percentage of volcanic ash increases, the results show tremendous improvement in the strength with the increase in lime content. The maximum value of strength was observed at lime and volcanic ash of 6% and 5%, respectively (Lime to blend ratio of 0.55). The UCS results are consistent with the results observed in Raman tests and laser microscopic images (Section 4.2). Raman results show that the materials in the volcanic ash have no cementing behavior unless mixed with lime with the presence of water, which in turn produces calcium silicate hydrates compounds (Figure 4b). In addition, the increase in lime content in the absence of volcanic ash as a source of pozzolans will not improve sand strength (Figure 4a). The results indicate that pozzolanic activities and thus the binding performance of the blend increases with the increase in volcanic ash and lime content, and the maximum increase in strength is observed at a lime-to-blend ratio of 0.55 (Lime 6% and VA 5%). This behavior is represented by the yellow shaded zone in Figure 4c, where the largest number of pozzolanic reaction compounds are formed. Furthermore, laser scanning microscopy images in Figure 9 show that for a lime-to-blend ratio of 0.55, large clusters bonded strongly with each other with a void-free and dense morphological structure forming large solidifications, are identified. As a result, the new integrated and compact structure is expected to have high compressive strength. Samples of the treated dune sand and after performing the UCS test were soaked in water, as shown in Figure 12. In Figure 12a it can be seen that the sample with lime: 6% and VA: 0% completely crumbled after a few minutes of soaking, while the one with lime: 6% and VA: 5% remained intact and strong for more than 60 days of soaking, as shown in Figure 12c. These results are consistent with the results obtained from the UCS tests.

Two factor multi-level statistical analysis were performed to find out the statistical significance of the results of the effects of lime and volcanic ash on UCS [54]. Coefficients of variance calculated for lime and volcanic ash treatments, using the nine test samples, on the UCS response are 13.8 and 6.9, respectively. The P values, which are the measure of confidence on the truth of the null hypothesis (H_o_), are calculated for both the effects of lime and VA on the UCS response. The statistically calculated P_lime_ and P_VA_ values for the UCS data are 0.001 and 0.0103, respectively. The P value for the lime is by an order of magnitude smaller than the traditional cutoff value for the meaningful statistical significance (≈0.05) and thus the UCS results are undoubtedly dependent on lime content. Whilst the P_VA_ value is smaller but comparable to the order of the statistical significance, implying a weak dependence of the UCS on the volcanic ash content.

### 4.4. Young’s Modulus (E_s_)

**Figure 13:**
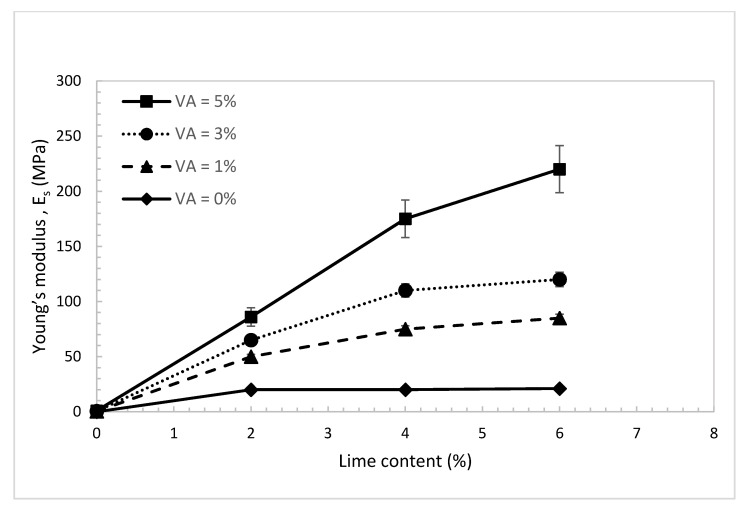
Young’s modulus of the treated dune sand vs. lime content for different percentages of volcanic ash.

Young’s modulus (E_s_) of the treated dune sand was also investigated at different percentages of lime and volcanic ash. The modulus was evaluated as the slope of the linear elastic region of the stress-strain curve. The results in Figure 13 show almost a similar trend and behavior to that observed in Figure 10 for the UCS. With the absence of volcanic ash, the improvement in E_s_ with the increase in lime content is insignificant. Besides that, there is almost no improvement in E_s_ for lime content greater than 2%. In addition, the results show that, with the absence of lime, there is no increase in E_s_ with the addition of volcanic ash. Ramon spectroscopy results in Figure 4a,b explained the observed behavior, which is directly related to the number of pozzolanic reaction compounds and the formation of dense and bonded clusters. As the percentage of the added volcanic ash increases, the results show a considerable increase in E_s_ with the increase in lime content. Interestingly, by using just 2% of lime and 1% of volcanic ash, sand E_s_ of the treated sand increases significantly from almost zero to 50 MPa. The slopes of the E_s_ curves in Figure 13 start to decrease for lime content greater than 2%. This means that for lime content greater than 2%, the effect of volcanic ash content on the E_s_ is more significant than the increase in the effect lime content.

Coefficients of variance calculated for lime and volcanic ash treatments, using the 9 test samples, on the Young’s modulus response are 5.1 and 6.0, respectively. The statistical analyses show that the calculated P_lime_ and P_VA_ values for the modulus are 0.0168 and 0.0068, respectively. Although the P_lime_ value is smaller but still comparable to the order of the statistical significance, implying a weak dependence of the modulus on lime content. On the contrary, the P_VA_ value is almost an order of magnitude smaller than the meaningful statistical significance (≈0.05) pointing towards the dependence of the modulus of the mix more so than on the volcanic ash.

### 4.5. California Bearing Ratio (CBR)

**Figure 14:**
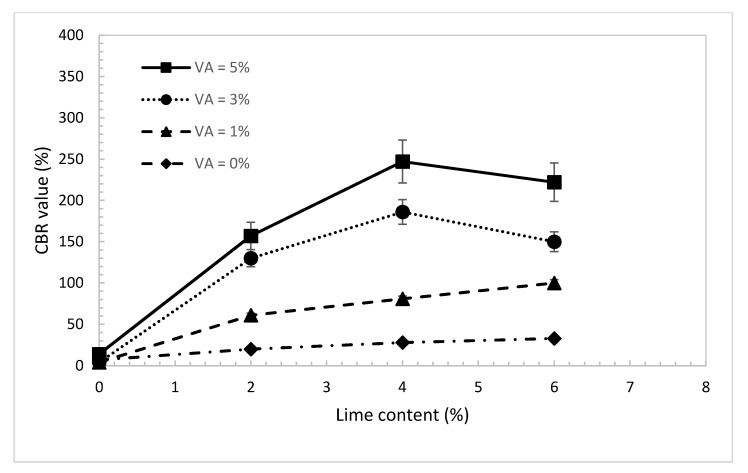
CBR value of the treated dune sand vs. lime content for different percentages of volcanic ash.

CBR tests (Figure 11b,c) were performed on un-soaked samples of the treated due sand at the maximum dry density and optimum water content of standard Proctor and for the same percentages of lime and volcanic ash used for the UCS tests. The results in Figure 14 show that with no use of volcanic ash, the CBR value increases slightly with the increase in lime content. As the percentage of the volcanic ash increases, the CBR value increases considerably. Apparently, the effect of volcanic ash on the CBR value is obvious even at low lime content. Using just 2% of lime and 3% of volcanic ash, the CBR value increases tremendously from 4% to 130%. As discussed before, and based on the results obtained from Raman and laser microscopy tests, pure lime mortar is not a suitable binder with the absence of the pozzolanic reaction compound. Moreover, the maximum number of reaction compounds are formed when lime and ash are blended almost in equal proportions (Lime/Blended ratio ≈0.55). Blends at too high or too law ratios will not complete the pozzolanic reaction due to the lack of sufficient reactants, resulting to low bearing resistance.

Coefficients of variance calculated for lime and volcanic ash treatments, using the 9 test samples, on the CBR response are 7.6 and 8.2, respectively. The statistical analyses show that the P_lime_ and P_VA_ values for the CBR are 0.0077 and 0.0059, respectively, which are much smaller than the traditional cutoff value for the meaningful statistical significance ≈0.05. Since the difference between the P values are also small, therefore the CBR of the mix equally depends on both the lime and VA.

### 4.6. Practical Relationships among UCS, E_s_, and CBR Value of the Treated Dune Sand

**Figure 15:**
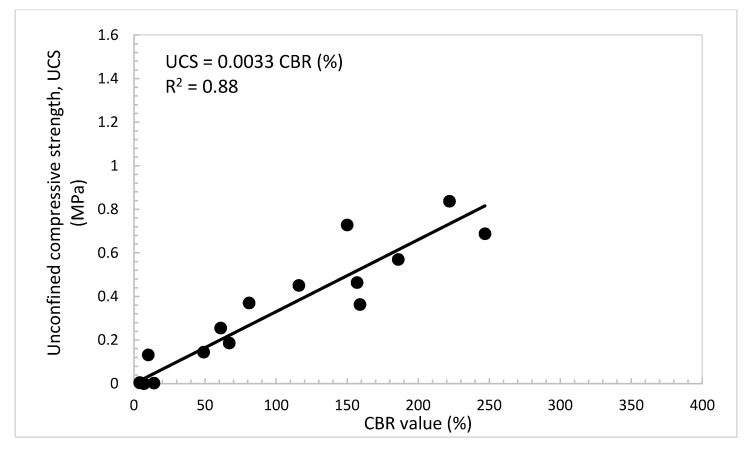
Unconfined compressive strength, UCS vs. CBR value of the treated dune sand.

**Figure 16:**
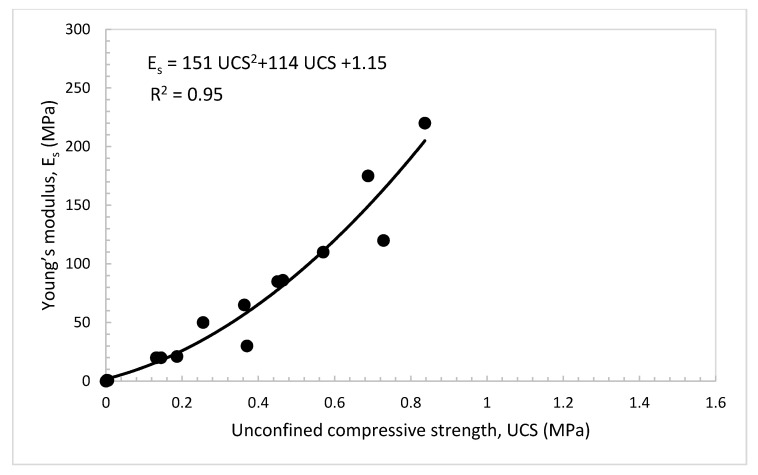
Young’s modulus, E_s_ vs. unconfined compressive strength of the treated dune sand.

**Figure 17:**
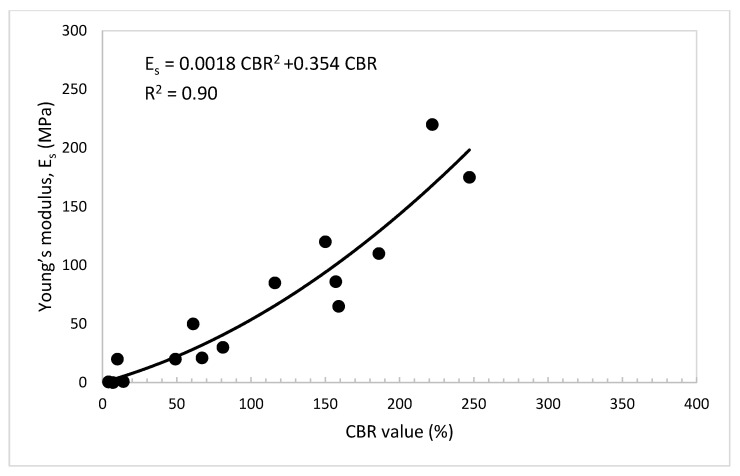
Young’s modulus, E_s_ vs. CBR value of the treated dune sand.

For engineering practice and to minimize the efforts of assessing the properties and parameters needed for the design of foundations of buildings and roadways, useful relationships were developed among the CBR value, UCS, and E_s_ of the dune sand treated with lime and volcanic ash, as presented in Figure 15, Figure 16 and Figure 17. Figure 15 shows that the UCS increases with the increase in the CBR value. The data show a good linear relationship (UCS = 0.0033 CBR (%)), with correlation factor R^2^ = 0.88. Figure 16 shows the relationship between the UCS and E_s_. In this figure, the E_s_ increases with the increase in the UCS and the relationship can be strongly expressed as a second-degree polynomial equation with correlation factor, R^2^ = 0.95. The relationship between the E_s_ and CBR value is presented in Figure 17. In this figure, it can be seen that the E_s_ also increases with the increase in the CBR value. The relationship can be expressed as a second-degree polynomial with a correlation factor, R^2^ = 0.90.

## 5. Conclusions

The testing program was conducted to investigate the effect of using lime and volcanic ash as stabilized materials on the engineering properties of dune sand used in the construction of foundations of roadways and buildings. The program consists of two major parts. The first part investigates the engineering properties of the treated sand, including UCS, Young’s modules, and CBR values. The second part focused on the chemical characteristic of the interaction between the additives using Raman spectroscopy and morphologic analysis of the lime-ash-sand mortar using laser-scanning microscopy (LSM). The results of the testing program of the second part were very important and strongly supported the findings obtained from the testing programs of the first one. The following conclusions were derived:

1. The Raman spectroscopy analysis of the samples having hydrated lime and volcanic ash with lime to blend ratio of 0.55 (VA = 6% and L = 5%) showed better Raman peaks as compared to all other samples. The high Raman peaks demonstrated high pozzolanic reactivity between lime and volcanic ash and ultimately formed a large number of reaction products for better strength properties;
2. Laser scanning microscopy (LSM) results showed that dune sand treated with hydrated lime and volcanic ash with lime to a blend ratio of 0.55 were more compacted and had better binding properties than other mixtures. High pozzolanic reactivity of both lime and volcanic ash resulted in the formation of reactive compounds which improved the adhesion between the sand particles and are ultimately responsible for the compact packing of sand particles;
3. The UCS and CBR value of the treated dune sand increased with the increase in the percentages of volcanic ash and lime content. The maximum value of strength was observed at a lime-to-blend ratio of 0.55. The UCS and CBR results are consistent with the results observed from Raman and laser microscopic tests for a lime-to-blend ratio of 0.55;
4. As the percentage of the added volcanic ash increased, the E_s_ of the treated dune sand increased with the increase in lime content. Interestingly, using just 2% of lime and 1% of volcanic ash, the E_s_ increased significantly from almost zero to 50 MPa;
5. Practical and useful relationships were developed among the CBR value, UCS, and E_s_ of the dune sand treated with lime and volcanic ash. These relations can helpfully be used for the design of foundations of roadways and structures using dune sands stabilized with lime and volcanic ash.

The current study concluded that the combined use of volcanic ash and hydrated lime were more effective in the stabilization of dune sand by improving its mechanical and microstructural properties. However, to further investigate the effective use of locally available VA as a potential dune sand stabilizer, future research is recommended by using the blend of VA with cement and other highly reactive materials such as silica fume and metakaolin for the stabilization of dune sand. To get a better understating of the properties of the treated dune sand with VA and reactive materials, other aspects may need to be considered such as the effect of water soaking in conjugation with curing time. This would help the researcher and designer in selecting the best stabilization materials for stronger, economical, and sustainable construction. The main challenge facing the use of treated dune sand as construction materials for roadways in Saudi Arabia is to set proper standards and codes that cover the entire processes, including the design phase, construction procedures and methods, monitoring techniques and tools, and remedial measure processes. This can be solved and improved with close cooperation between researchers in this field and the decision makers of the roadways industry.

## Figures and Tables

**Table 1:** Summary of the physical properties and classification of the dune sand.

Soil Property	Value/Description
Specific Gravity [30]	2.68
Color	Yellow
D_10_ (mm)	0.18
D_30_ (mm)	0.3
D_60_ (mm)	0.42
C_C_	1.2
C_u_	2.3
Shape of Particles	Both, coarse and fine portions are rounded and sub rounded in shape
Classification-AASHTO system	Non-plastic fine sand (A3)
Classification-USCS system	Poorly graded sand (SP)

**Table 2:** Chemical composition of volcanic ash, VA [31].

Oxide	% by Weight
SiO_2_	46.4
Al_2_O_3_	14.4
Fe_2_O_3_	12.8
CaO	8.8
MgO	8.3
Na_2_O	3.8
K_2_O	1.9
SO_3_	0.8
LOI (Loss on ignition)	2.8

**Table 3:** Physical and engineering testing program of the treated dune sand.

Test	Percentage of Lime	Percentage of Volcanic Ash	Curing Time (Days)	Used Standard
Specific gravity	0	0	-	ASTM D854 [30]
Grain size analysis	0	0	-	ASTM D6913 [27]
Standard proctor compaction	0	0	-	ASTM D698-07 [33]
Material classification (USCS). Material classification (AASHTO)	0	0	-	ASTM D2487-17 [28] AASHTO M 145-82 [29]
Unconfined compressive strength (UCS)	0, 2, 4, 6	0, 1, 3, 5	28	ASTM D2166-85 [34] (Method A)
California bearing ratio (CBR)	0, 2, 4, 6	0, 1, 3, 5	28	ASTM D1883-07 [35] (Method C)

**Table 4:** Tested samples for Raman spectroscopy and laser-scanning microscopy (LSM).

Sample Number (#)	Sample Type and Percentage Used	Lime/Blend Ratio (L/L + VA)
a	(L:VA)—(0:5)	0.0
b	(L:VA)—(6:0)	1.0
c	(L:VA)—(6:1)	0.86
d	(L:VA)—(2:1)	0.67
e	(L:VA)—(6:5)	0.55
f	(L:VA)—(2:3)	0.40

L: Lime, VA: Volcanic Ash.

## Data Availability

The data presented in this study are available on request from the corresponding author.

## Abbreviations

A3	Non-plastic fine sand
AASHTO	American Association of State Highway and Transportation Officials
ASTM	American Society for Testing and Materials
A.U.	Airy unit
CBR	California Bearing Ratio
C_c_	Coefficient of curvature
C_u_	Coefficient of uniformity
C-A-H	Calcium aluminate hydrate
C-S-H	Calcium silicate hydrate
C_3_A	Tricalcium aluminate
C_4_AF	Tetracalcium alumino ferrite
C_2_S	Tricalcium silicate
E_s_	Young’s modulus
D_10_	Grain diameter (in mm) corresponding to 10% passing
D_30_	Grain diameter (in mm) corresponding to 30% passing
D_50_	Grain diameter (in mm) corresponding to 50% passing
D_60_	Grain diameter (in mm) corresponding to 60% passing
D_90_	Grain diameter (in mm) corresponding to 90% passing
GGBFS	Ground granulated blast furnace slag
GSD	Grain Size Distribution
L	Lime
LOI	Loss on ignition
LSM	Laser-scanning microscopy
P_lime_	Factor of multi-level statistical analysis for lime
P_VA_	Factor of multi-level statistical analysis for volcanic ash
ppm	Parts per million
SP	Poorly graded sand
SP-SM	Poorly graded sand with silt
UCS	Unconfined compressive strength
USCS	Unified Soil Classification System
VA	Volcanic ash
XRD	X-ray diffraction
